# Mutants in the lipopolysaccharide of *Brucella ovis* are attenuated and protect against *B. ovis* infection in mice

**DOI:** 10.1186/s13567-014-0072-0

**Published:** 2014-07-17

**Authors:** Pedro Soler-Lloréns, Yolanda Gil-Ramírez, Ana Zabalza-Baranguá, Maite Iriarte, Raquel Conde-Álvarez, Amaia Zúñiga-Ripa, Beatriz San Román, Michel S Zygmunt, Nieves Vizcaíno, Axel Cloeckaert, María-Jesús Grilló, Ignacio Moriyón, Ignacio López-Goñi

**Affiliations:** 1 Departamento de Microbiología y Parasitología and Instituto de Salud Tropical, Universidad de Navarra, Pamplona, 31008, Spain; 2 Instituto de Agrobiotecnología (CSIC-Universidad Pública de Navarra-Gobierno de Navarra), Pamplona, 31006, Spain; 3 INRA, UMR1282 Infectiologie et Santé Publique, Nouzilly, F-37380, France; 4 Université François Rabelais de Tours, UMR1282 Infectiologie et Santé Publique, Tours, F-37000, France; 5 Departamento de Microbiología y Genética, Universidad de Salamanca, and Instituto de Investigación Biomédica de Salamanca (IBSAL), Salamanca, Spain

## Abstract

*Brucella* spp. are Gram-negative bacteria that behave as facultative intracellular parasites of a variety of mammals. This genus includes smooth (S) and rough (R) species that carry S and R lipopolysaccharides (LPS), respectively. S-LPS is a virulence factor, and mutants affected in the S-LPS O-polysaccharide (R mutants), core oligosaccharide or both show attenuation. However, *B. ovis* is naturally R and is virulent in sheep. We studied the role of *B. ovis* LPS in virulence by mutating the orthologues of *wadA*, *wadB* and *wadC*, three genes known to encode LPS core glycosyltransferases in S brucellae. When mapped with antibodies to outer membrane proteins (Omps) and R-LPS, *wadB* and *wadC* mutants displayed defects in LPS structure and outer membrane topology but inactivation of *wadA* had little or no effect. Consistent with these observations, the *wadB* and *wadC* but not the *wadA* mutants were attenuated in mice. When tested as vaccines, the *wadB* and *wadC* mutants protected mice against *B. ovis* challenge. The results demonstrate that the LPS core is a structure essential for survival in vivo not only of S brucellae but also of a naturally R *Brucella* pathogenic species, and they confirm our previous hypothesis that the *Brucella* LPS core is a target for vaccine development. Since vaccine *B. melitensis* Rev 1 is S and thus interferes in serological testing for S brucellae, *wadB* mutant represents a candidate vaccine to be evaluated against *B. ovis* infection of sheep suitable for areas free of *B. melitensis*.

## Introduction

Brucellosis is a worldwide extended infectious disease caused by the Gram-negative bacteria of the genus *Brucella*. This genus includes several species among which *B. abortus* preferentially infects cattle, *B. suis* swine and wild-life and *B. melitensis* goats and sheep. These three species are zoonotic and cause a grave and debilitating disease in humans. Sheep can also be infected by *B. ovis*, a non-zoonotic species. *B. ovis* brucellosis is characterized by a decreased fertility in rams, occasional abortions and a rise in perinatal mortality [[1],[2]]. These four *Brucella* species differ not only in host range and pathogenicity but also in surface characteristics. Whereas *B. abortus*, *B. melitensis* and *B. suis* carry a smooth (S) type lipopolysaccharide (LPS) in the outer membrane, *B. ovis* LPS lacks the O-polysaccharide typical of S-LPS and thus resembles in this feature the rough (R) LPS mutants of S brucellae [[3]].

**Figure 1:**
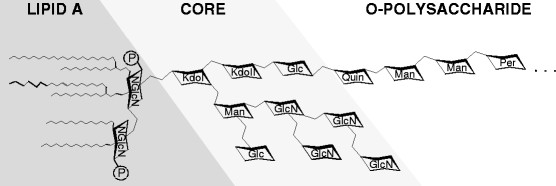
**Proposed structure of the** ***Brucella*** **core LPS as described by Kubler-Kielb [**[8]**].** The core oligosaccharide is composed of a side oligosaccharide chain composed of four residues of glucosamine (GlcN), plus glucose (Glc) and mannose (Man), the latter linked to Kdo I, with Kdo II linked to another glucose from which the O-polysaccharide stems. Quin: quinovosamine; Per: perosamine.

S-LPS is a major virulence factor of S *Brucella* species [[4]]. In this molecule, the O-polysaccharide is linked to a core oligosaccharide, which in turn is linked to the lipid A. It has been known for decades that the O-polysaccharide is essential in the virulence of *B. abortus*, *B. melitensis* and *B. suis,* and that the lipid A is poorly recognized by innate immunity [[4]]. In addition, the core oligosaccharide section has been shown recently to hamper recognition by innate immunity systems, including complement, bactericidal peptides and the TLR4-MD2 complex [[5]]. It has been postulated that the *Brucella* S-LPS core carries a lateral branch that hinders access of innate immunity effector proteins and receptors to the inner sections of the core and lipid A [[5]–[7]], and the existence of a branched structure has been confirmed by structural analysis (Figure 1) [[8]]. These findings have opened the way for an analysis of the role of the LPS of R *Brucella* species in virulence. Moreover, as delayed recognition by innate immunity plays a major role in *Brucella* virulence, core mutants represent candidates for the development of vaccines triggering an early and thus protective immunoresponse [[6]].

Sheep brucellosis caused by either *B. melitensis* or *B. ovis* can be controlled by vaccination with the attenuated strain *B. melitensis* Rev 1 and, in fact, this is the only effective way to control sheep brucellosis in areas with a high or moderate prevalence of the disease [[9]]. However, Rev 1 has several drawbacks: it causes an antibody response interfering with the serological diagnosis of *B. melitensis*, is virulent in humans and resistant to streptomycin, an antibiotic of choice for brucellosis treatment [[9]]. Therefore, Rev 1 is forbidden in countries where *B. melitensis* has been eradicated [[9]], which leads to the increase of *B. ovis* infections in sheep. Thus, research on *B. ovis*-specific vaccines is an area of intense research [[10]–[12]]. Acellular vaccines can be an alternative, and attempts have been carried out with whole cell and subcellular extracts [[13]], recombinant proteins [[14]] or DNA vaccines [[15]]. However, these strategies usually require boosters and adjuvants or immune modulators to reach an adequate Th1 response [[10],[16]], with the ensuing increase in costs and difficulties of implementation in extensively bred sheep.

The purpose of the research reported here was twofold: to study the involvement of LPS in *B. ovis* virulence and to develop *B. ovis*-specific vaccine candidates that could overcome the above-summarized drawbacks. To this end, we built on previous genetic analyses that have shown that at least three glycosyltransferases (*wadA*, *wadB* and *wadC*) are necessary for the complete assembly of the core oligosaccharide of *B. abortus* and *B. melitensis* [[5],[7],[17],[18]].

## Materials and methods

### Bacterial strains and growth conditions

The bacterial strains and plasmids used in this work are listed in Table 1. The parental strain *B. ovis* PA is a virulent strain isolated from a naturally infected ram that has been extensively used as a challenge for the evaluation of *B. ovis* vaccines in rams and mice. *B. ovis* strains were cultured on tryptic soy agar (TSA, Pronadisa, Madrid, Spain) or in tryptic soy broth (TSB, Biomerieux, Madrid, Spain) supplemented with 0.5% yeast extract (YE, Merck, Madrid, Spain) or on Blood Agar Base No. 2 (BAB; Pronadisa), all supplemented with 5% porcine or calf serum (TSA-YE-S, TSB-YE-S or BAB-S, respectively). Incubations were performed at 37 °C in a 10% CO_2_ atmosphere, and liquid cultures were shaken at low intensity. *E. coli* was grown in Luria-Bertani broth (LB: Becton Dickinson, Madrid, Spain). Nalidixic acid (Nal; 25 μg/mL), Kanamycin (Km; 50 μg/mL), Gentamicin (Gm; 15 μg/mL) or sucrose (5% w/v) (all from Sigma-Aldrich Ltd., Haverhill, United Kingdom) were used when required.

**Table 1:** Bacterial strains and plasmids used in this work

**Bacterial strains/Plasmids**	**Characteristic**	**Source/Reference**
***Brucella ovis***		
PA	virulent strain, natural Nal^R^	CITA collection
BoΔ*wadA*	PA in frame deletion mutant in *wadA* Δ58-679	This work
BoΔ*wadB*	PA in frame deletion mutant in *wadB* Δ49-195	This work
BoΔ*wadC*	PA in frame deletion mutant in *wadC* Δ17-306	This work
BoPA-Gm	challenge strain, PA Gm^R^	UN collection
***Brucella melitensis***		
Rev 1	reference vaccine	CITA collection
***E. coli***		
S17-1 λpir	mating strain with plasmid RP4 inserted into the chromosome	Simon et al. [[19]]
TOP10 F’	F^−^*lacI*q Tn *10* (Tet^R^) mcrA Δ(*mrr-hsdRMS-mcrBC*) 80*lacZ* ΔM15 Δ*lacX74 recA1alaD139 endA1 nupG*	Invitrogen
**Plasmids**		
pCR2.1	cloning vector	Invitrogen
pJQK	derivated of pJQ200KS+; Km^R^, Gm^S^	Scupham and Triplett [[20]]
pYRI-12	913-bp of *B. abortus* parental chromosomal DNA containing the *wadA* deletion allele, generated by PCR and cloned into pCR2.1	This work
pYRI-13	*BamHI-XbaI* fragment from pYRI-12 cloned into the corresponding sites of pJQK	This work
pYRI-1	570-bp of *B. abortus* parental chromosomal DNA containing the *wadB* deletion allele, generated by PCR and cloned into pCR2.1	Gil-Ramírez et al. [[7]]
pYRI-2	*BamHI-XbaI* fragment from pYRI-1 cloned into the corresponding sites of pJQK	Gil-Ramírez et al. [[7]]
pYRI-14	934-bp of *B. ovis* parental chromosomal DNA containing the *wadC* deletion allele, generated by PCR and cloned into pCR2.1	This work
pYRI-15	BamHI-XbaI fragment from pYRI-14 cloned into the corresponding sites of pJQK	This work

CITA, Centro de investigación y tecnología agroalimentaria; UN, Universidad de Navarra.

### DNA manipulations and sequence analyses

Plasmid and genomic DNA were isolated with Qiaprep Miniprep (Qiagen GmbH, Hilden, Germany) and Ultraclean Microbial DNA Isolation Kit (MoBio Laboratories, Carlsbad, CA, USA), respectively. When needed, DNA was also purified from agarose gels using a Qiack Gel extraction kit (Qiagen). DNA sequencing was performed by the dideoxy method at the Sequencing Unit of Centro de Investigación Médica Aplicada (CIMA, Universidad de Navarra, Spain), and primers were synthesized by Sigma-Aldrich Ltd. Searches for DNA and protein homologies were carried out using the Kyoto Encyclopedia of Genes and Genomes [[21]], EMBL-European Bioinformatics Institute server [[22]] and National Center for Biotechnology Information (NCBI) database [[23]].

### Construction of LPS mutants

In-frame deletion mutants on selected genes were constructed by PCR overlap using genomic DNA of *B. ovis* PA as a DNA template. Primers were designed based on the sequence of *B. ovis* ATCC 25840 (also known as 63/290 or NCTC10512; accession numbers NC_009505.1 and NC_009504.1). They are listed in Table 2. For inactivation of *wadC* (BOV_1453), we first generated two PCR fragments: the 5′end of the gene BOV_1453 was amplified with primers *wadC*-F1 and *wadC*-R2 obtaining a 473-bp fragment including codons 1 to 16 of the *wadC* ORF, as well as 424-bp upstream of the *wadC* start codon; whereas the 3′end was amplified with primers *wadC*-F3 and *wadC*-R4 obtaining a 481-bp fragment including codons 307 to 355 of the *wadC* ORF and 315-bp downstream of the *wadC* stop codon. Both fragments were ligated by overlapping PCR using primers *wadC*-F1 and *wadC*-R4. The resulting fragments containing the *wadC* deletion allele, was cloned into pCR2.1 vector to generate plasmids pYRI-14 (Table 1), sequenced to ensure the maintenance of the reading frame, and subsequently subcloned into the *BamH*I and the *Xba*I sites of the suicide plasmid pJQK. The resulting mutator plasmid (pYRI-15, Table 1) was introduced in *B. ovis* PA by conjugation using *E. coli* S17-1λpir. The first recombination event (integration of the suicide vector in the chromosome) was selected by Nal and Km resistance, and the second (excision of the mutator plasmid leading to the deletion mutant strain by allelic exchange) by Nal and sucrose resistance and Km sensitivity. The resulting colonies were screened by PCR with primers *wadC*-F1 and *wadC*-R4, which amplify a fragment of 934-bp in the mutant strain and a fragment of 1804-bp in the parental strain. An additional PCR was carried out to exclude the presence of the complete gene. The amplification was done with primers *wadC*-F1 and *wadC*-R5, which include a fragment from the deleted region of the gene. While strains carrying the complete gene amplified a 533-bp fragment, the mutant strain was unable to amplify the fragment (see Additional file 1). The deletion was confirmed by sequencing. As a result of the mutation 82% of the *wadC* ORF was lost, and the mutant strain was called BoΔ*wadC* (Table 1).

**Table 2:** Primers used in the study

**Gene**		**Sequence (5’-- > 3’)**
*wadA*	F1	CCC ACG CTG CTT AGT ACG TT
	R2	CAT CAA AAC GTG CAT CGT CAA
	F3	ATT GAC GAT GCA CGT TTT GAT GCA TTC GGC TTT GCC TTT TAT
	R4	GAG TTT ATC GCC CAA TTT GC
	R5	TCT TCC AGA ATG AGG CCG TA
*wadB*	F1	GCA TGA TTA CCC CGC TGAT
	R2	CGC AAT CTC GTC TTT GTT GAG
	F3	CTC AAC AAA GAC GAG ATT GCG GGT GGC GTG AAG GAA ATCT
	R4	TGA TAG CCG AGC CTC TTC AG
	R5	ATG CAC CCA TGA AGT TTT CC
*wadC*	F1	CTG GCG TCA GCA ATC AGA G
	R2	GTG CAA CGA CCT CAA CTT CC
	F3	GGA AGT TGA GGT CGT TGC ACA CGC CAT CGA ACC TTA TCT G
	R4	CGG CTA TCG TGC GAT TCT
	R5	GCA ATG GAA TGA GCT GAA CA

The *ΔwadA* mutant (BOV_0614) was obtained in a similar way. Briefly, primers *wadA*-F1 and *wadA*-R2 were used to amplify a 453-bp fragment including codon 1 to 57 of the 5′end of *wadA* (BOV_0614), as well as 284-bp upstream of the *wadA* start codon; and primers *wadA*-F3 and *wadA*-R4 were used to amplify a 460-bp fragment including codons 680 to 704 of the *wadA* ORF and 386-bp downstream of the *wadA* stop codon. Both fragments were ligated by overlapping PCR using primers *wadA*-F1 and *wadA*-R4, the resulting fragment was cloned into pCR2.1, sequenced and subcloned into pJQK. The resulting mutator plasmid (pYRI-13, Table 1) was introduced in *B. ovis* PA by electroporation with a micropulser (Bio-Rad, Hercules, CA, USA) as described before [[24]]. The first recombination event was selected by Km resistance, and the second by sucrose resistance and Km sensitivity. The resulting colonies were screened by PCR with primers *wadA*-F1 and *wadA*-R4, which amplify a fragment of 913-bp in the mutant strain and a fragment of 2782-bp in the parental strain. An additional PCR was carried out to exclude the presence of the complete gene. The amplification was done with primers *wadA*-F1 and *wadA*-R5, which include a fragment from the deleted region of the gene. While strains carrying the complete gene amplified a 565-bp fragment, no amplification was obtained with the mutant strain (see Additional file 1). The deletion was confirmed by sequencing. As a result of the mutation 82% of the *wadA* ORF was lost and the mutant strain was called BoΔ*wadA* (Table 1).

Taking into account that the *wadB* sequence of *B. abortus* 2308 and *B. ovis* PA are almost identical (99.86% homology with only one base pair change in the deleted section of the gene), we used the mutator plasmid pYRI-2 (Table 1) that was previously constructed to generate Δ*wadB* (BAB1_0351) mutants in *B. abortus* 2308 for the construction of the *B. ovis* Δ*wadB* mutant [[7]]. The mutator plasmid pYRI-2 was introduced in *B. ovis* PA by conjugation. The resulting colonies were screened by PCR with primers *wadB*-F1 and *wadB*-R4, which amplify a fragment of 570-bp in the mutant strain and a fragment of 1011-bp in the parental strain. An additional PCR was carried out to exclude the presence of the complete gene. The amplification was done with primers *wadB*-F1 and *wadB*-R5, which include a fragment from the deleted region of the gene. While strains carrying the complete gene amplified a 471-bp fragment, the mutant strain was unable to amplify the fragment (Additional file 1). The deletion was confirmed by sequencing. As a result of the mutation 60% of the *wadB* ORF was lost, and the mutant strain was called BoΔ*wadB* (Table 1).

In all mutagenesis experiments, after the second recombination, two type of colonies Nal and sucrose resistant and Km sensitive could be isolated: the deletion mutant strain and the strain that had reverted to the wild-type genotype (hereafter “sibling revertant strain”). The latter was also selected as control for additional mutations elsewhere in the chromosome that may have been acquired during the manipulations described above, and the genotypes of all the strains were confirmed by PCR amplification and sequencing of the target locus in the genome. Moreover, both mutant and sibling revertant strains were characterized following standard *Brucella* typing procedures [[25]], and purity checked by inhibition of growth on BAB-S supplemented with Km.

### LPS extraction and characterization

Extraction of whole-cell LPS by SDS-proteinase K protocol was performed as described by Dubray and Limet [[26]] with modifications. Briefly, heat inactivated cells were suspended in 0.0625 M Tris–HCl buffer (pH 6.8) containing 2% SDS (wt/wt). Samples were heated at 100 °C for 10 min, and the lysates were tempered to 55 °C and digested twice with proteinase K (10 mg/mL, one hour at 55 °C). After that, samples were kept overnight at 20 °C and then stored at −20 °C. LPS were analysed in Tris-Tricine-HCl-glycine poliacrilamyde gel electrophoresis (Tricine SDS-PAGE) as described by Lesse [[27]]. Briefly, a 16 × 20 cm two-phase gel (18% acrylamide-bisacrylamide for the running gel and 4% for the stacking gel) was prepared in 1 M Tris 0.1% SDS, pH 8.45. The gel was placed between anode and cathode buffers (0.2 M Tris–HCl pH 8.9 and 0.2 M Tris–HCl, 0.1 M Tricine, 0.1% SDS pH 8.45, respectively) and the electrophoresis was carried out at constant voltage for one h at 30 V followed by 20 h at 70 V. LPS was stained by the periodate-alkaline silver method [[28]]. For Western blots, LPS was electrotransferred onto nitrocellulose membranes, blocked with 1% skimmed milk in PBS for 45 min and incubated with specific monoclonal antibodies (MAbs, see below) overnight. After washing, bound immunoglobulins were detected by chemiluminiscence (Thermo Fisher Scientific Inc., Waltham, USA) with goat anti mouse IgG (H + L) chain specific *B. ovis* conjugate secondary antibodies (Merk4Biosciencies, Damstadt, Germany).

### Surface topology mapping

Outer membrane proteins (Omp) and LPS exposure on the cell surface was assessed by ELISA with the following MAbs: A76/08C03/G03 for Omp16, A76/05C10/A08 for Omp19, A68/25G05/A05, A68/15B06/C08 and A63/04D11/G01 for Omp2b, A59/05F01/C09 for Omp25, A59/10F09/G10 for Omp31, and A68/24G12/A08, A68/24D08/G09 and A68/10A06/B11 for R-LPS [[29]–[32]]. ELISA using whole bacteria as the antigen were performed as described previously [[32],[33]] with some modifications. Briefly, lyophilized bacteria were resuspended up to OD_600_ 1 in phosphate-buffered saline pH 7.4 (PBS). For coating, this suspension was distributed (100 μL/well) in 96 flat bottom plates (Thermo Scientific) and incubated overnight at room temperature. One-hundred microliters of MAbs (hybridoma supernatant diluted 1:3 in 0.05% Tween PBS) were incubated 37 °C for 1 h. Bound MAbs were then detected with an affinity-purified goat anti-mouse immunoglobulin G (H + L chains) – horseradish peroxidase conjugate (Bio-Rad, Hercules, CA, USA) (diluted 1:6000 in 0.05% Tween PBS) incubated for 1 h at room temperature. Between all the previous steps five washes with 250 μL 0.05% Tween PBS were performed in order to eliminate the excess of MAbs and conjugate. Enzyme activity was revealed by the addition of TMB ELISA peroxidase substrate (Interchim, Montlucon, France) and stopped after 20 min at room temperature with 1 M HCl based stop solution (Interchim). Plates were read at 450 nm with a microplate reader (Thermo Fisher Scientific Inc.).

### Physico-chemical surface properties

Autoagglutination capacity was evaluated by measuring the OD_600_ evolution of a bacterial suspension in TSB-YE-S, over 48 h of static incubation at room temperature, starting from initial readings of 0.8 (100% OD_600_) in TSB-YE-S [[24]]. The results were represented as the mean ± SD of the values obtained for three assays at each time point, in three independent assays. The surface charge density was measured as the electrophoretically effective potential (Zeta potential) [[18]]. For this, bacteria were inactivated with 0.5% phenol, washed and resuspended in 0.1 M KCl, 10 mM HEPES 10 mM (pH 7.2) at an OD_600_ of 0.1. Measurements were performed at 25 °C in a Zetamaster instrument using the PCS 1.27 software (Malvern Instruments Ltd., Malvern, UK) and the settings of aqueous solutions (viscosity = 1.002 cP; dielectric constant = 80.4), in plain buffer.

### Susceptibility to nonimmune serum and polymyxin B

Sensitivity to the bactericidal action of nonimmune serum was determined as follows: exponentially growing bacteria were adjusted to 2 × 10^4^ CFU/mL in PBS and dispensed in microtiter plates (50 μL/well) containing fresh normal calf serum or serum previously heated at 56 °C for 60 min to remove complement (150 μL/well). After incubation for 3 h at 37 °C, 50 μL of each sample was plated TSA-YE-S by triplicate, and the results are expressed as the proportion between the organism cultivable after exposure to normal nonimmune serum and those cultivable after the exposure to serum in which complement had been heat inactivated. Sensitivity to polymyxin B (Sigma-Aldrich Ltd.) was determined as described before [[24]].

### Virulence and vaccine efficacy studies in mice

Female 8–10 week-old BALB/c mice (Charles River International, France) were used. They were randomly distributed and accommodated in the animal facilities of Universidad Pública de Navarra (registration code ES/31-2016-000002 CR-SU-US) for 1–2 weeks before starting and during the assays, with water and food *ad libitum.* The procedures performed with mice were designed according to national (RD 53/2013) and European (EU directive 2010/63) legislations regarding the use of animals in research. Mice inoculations were carried out with 0.1 mL of bacterial suspensions previously adjusted to an OD_600_ of 0.170 (around 1 × 10^9^ CFU/mL) and then diluted to the appropriate dose (see below) in sterile PBS [[34]]. Exact inoculation doses were determined retrospectively by plating tenfold dilutions prepared in PBS on BAB-S or TSA-YE-S.

For virulence, BALB/c mice (*n* = 10) were inoculated intraperitoneally (IP) with 5–7 × 10^5^ CFU/mouse of the corresponding *B. ovis* PA mutant strain, and viable spleen counts were determined (*n* = 5) at 3 and 10 weeks post-inoculation as previously detailed [[34]]. As a control, additional groups of mice (*n* = 10) were inoculated similarly with the virulent *B. ovis* PA reference or the sibling reverting strains, to control for the potential for incidental attenuation during the manipulation required for mutagenesis. The identity of the spleen isolates was confirmed throughout the experiment by both PCR and *B. ovis* phenotypic features [[25]]. Spleen infections were expressed as mean ± SD (*n* = 5) of individual log_10_ CFU/spleen at the times indicated [[34]].

Efficacy of the BoΔ*wadB* and BoΔ*wadC* mutants as vaccines was evaluated in BALB/c mice (*n* = 10) vaccinated intraperitoneally with 1 × 10^8^ CFU/mouse of the corresponding *B. ovis* mutant strain, or subcutaneously (SC) with 1 × 10^5^ CFU/mouse of *B. melitensis* Rev 1 (as standard vaccinated control), or with 0.1 mL of PBS (pH 6.8) as the unvaccinated control. Four weeks after vaccination, all mice were challenged intraperitoneally with 5 × 10^5^ CFU/mouse of the virulent *B. ovis* PA-Gm^R^ (Table 1), and the number of challenge bacteria in spleens was determined 3 weeks afterwards [[35]]. Differentiation between challenge and residual vaccine bacteria was performed by duplicate plating on BAB-S and BAB-S supplemented with Gm. Results are expressed as the mean and SD (*n* = 10) of the individual log_10_ CFU/spleen of *B. ovis* PA-Gm^R^ challenge strain. Statistical comparisons of mean log_10_ CFU/spleen were performed by a one-way ANOVA followed by the Fisher’s Protected Least Significant Differences tests [[34]]. The virulence of the *B. ovis* PA-Gm^R^ challenge strain used in this work was proved in a previous experiment in BALB/c mice (*n* = 5) by intraperitoneal infection (5 × 10^5^ CFU/mouse) and bacterial spleen counting at 3 weeks later. This strain showed identical bacterial counts in BAB-S and BAB-S supplemented with Gm, and identical multiplication to that of *B. ovis* PA in mice (not shown).

## Results

### Construction of *B. ovis* LPS mutants

Several ORF encoding putative glycosyltransferases have been shown to be involved in the biosynthesis of the core of *B. abortus* LPS: *wadA* (formerly named wa**, BAB1_0639, [[17],[18]]), *wadB* (BAB1_0351, [[6],[7]]) and *wadC* (BAB1_1522, [[5],[6]]). We searched for orthologues of these glycosyltransferases in the *B. ovis* strain ATCC25840 genome sequence and we identified three ORF: BOV_0614 for *wadA*, BOV_0337 for *wadB* and BOV_1453 for *wadC*, with 99.4%, 99.9% and 100% DNA sequence identity, respectively. Non-polar mutant and sibling revertant strains were obtained for each target gene from *B. ovis* PA strain as described in the Materials and methods section. PCR amplification and sequencing of the target loci in the genome of the mutants confirmed inactivation of the respective genes (data not shown). All mutants behaved like the parental strain in classical bacteriological typing for the genus *Brucella* [[25]] and showed no differences in growth patterns when compared to the parental strain *B. ovis* PA (data not shown).

### *B. ovis wadB* and *wadC* are required for the synthesis of a complete LPS

**Figure 2:**
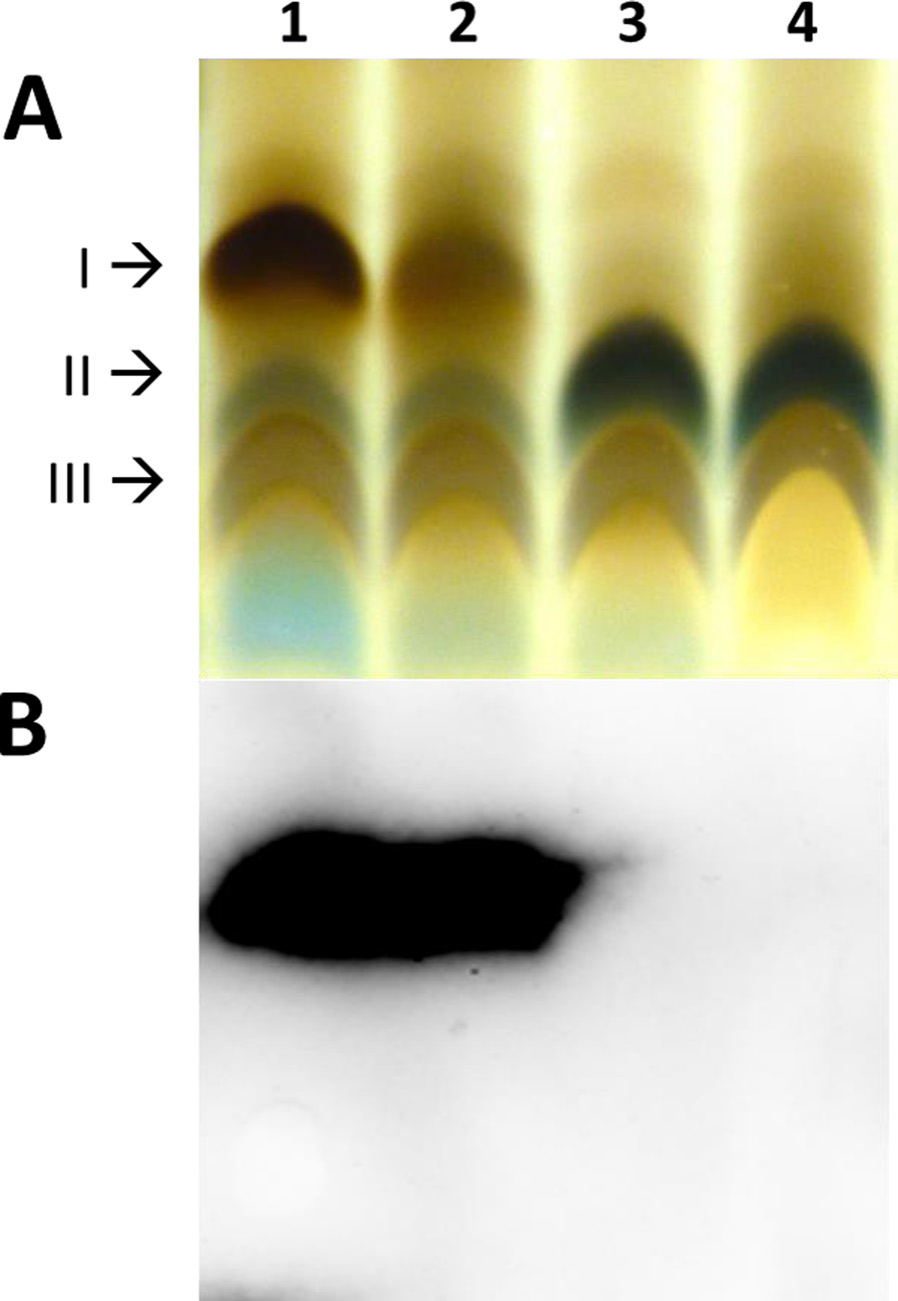
**LPS characterization.** Tricine SDS-PAGE **(A)** and Western-blot **(B)** analysis of the LPS of *B. ovis* PA (1) and the LPS mutants BoΔ*wadA* (2), BoΔ*wadB* (3) and BoΔ*wadC* (4). Arrows point the components I, II and III found by migration in the gel. The antibody used in the blot shown **(B)** is anti R-LPS A68/10A06/B11. Western blot probed with A68/24G12/A08, and A68/24D08/G09 gave similar results (data not shown).

For the analysis of possible LPS defects, we extracted *B. ovis* LPS using a SDS-proteinase K protocol [[26]]. Tricine SDS-PAGE resolved the LPS of the parental strain *B. ovis* PA into at least three major components I, II and III (Figure 2A), and the same migration pattern was obtained with the BoΔ*wadA* mutant and the sibling revertant strain (not shown). However, component I was absent from LPS of mutants BoΔ*wadB* and BoΔ*wadC*, which also showed increased relative proportions of component II, suggesting a deficiency in the LPS oligosaccharide structure. The corresponding blots were probed with anti-R-LPS MAbs A68/10A06/B11, A68/24G12/A08, and A68/24D08/G09 (Figure 2B). The lack of reactivity of these MAbs with the LPS of mutants BoΔ*wadB* and BoΔ*wadC* confirmed the absence of part of the oligosaccharide. In contrast, no defect could be detected in the LPS of mutant Bo∆*wadA* by this method.

### LPS gene mutants of *B. ovis* have altered key topological, physicochemical and biological surface properties

**Figure 3:**
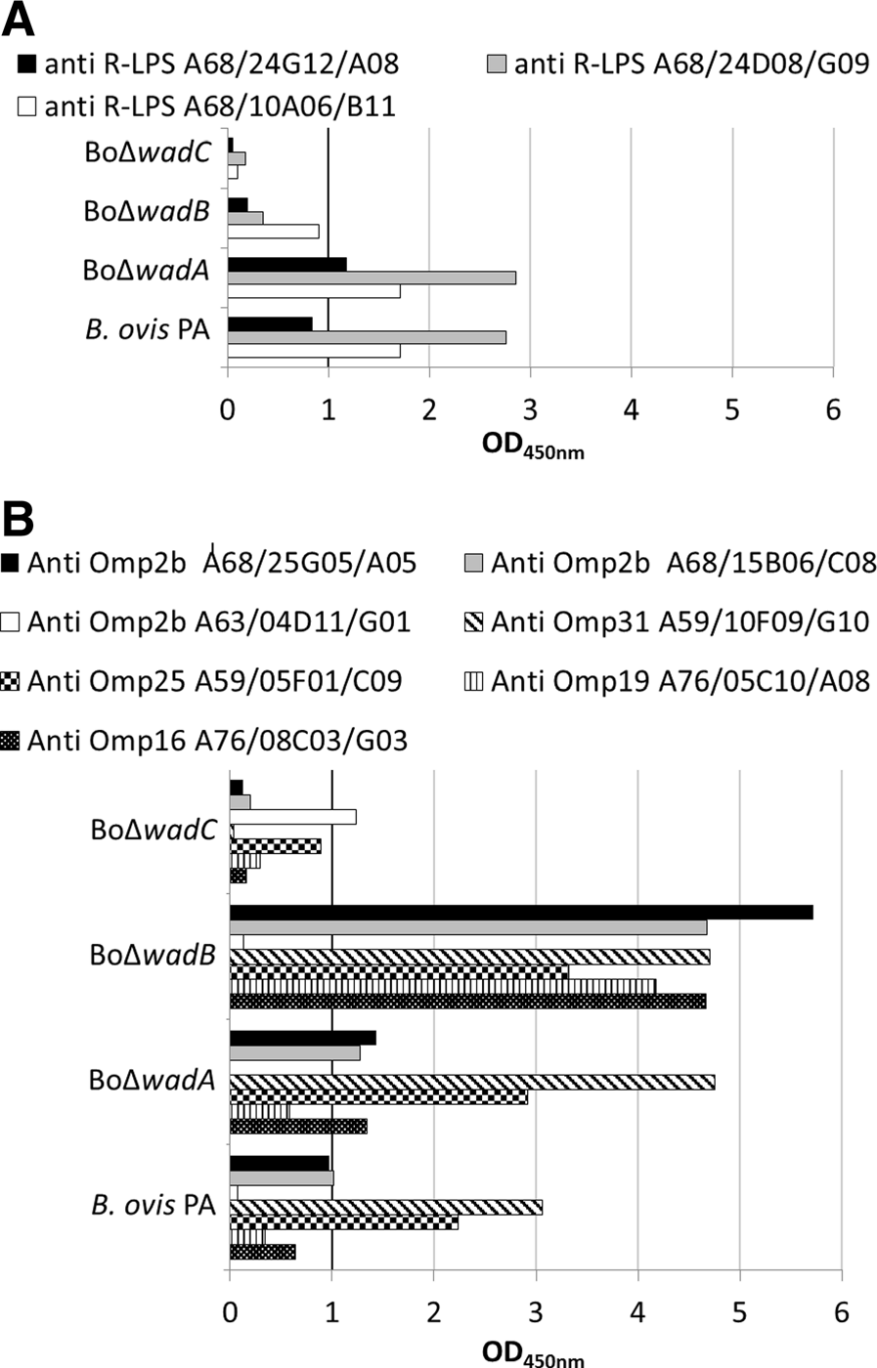
**Outer membrane epitopes in** ***B. ovis*** **PA and the LPS mutants.** Reactivity with MAbs anti R-LPS **(A)** and anti Omps **(B)** was measured by ELISA.

Several differences were found between two *B. ovis* LPS gene deletion mutants and the parental strain with regard to their reactivity with MAbs specific for R-LPS and Omps. When probed with anti-R-LPS antibodies, differences in reactivity suggestive of changes in epitopic structure and/or exposure were found: mutants BoΔ*wadB* and BoΔ*wadC* showed decreased reactivity while mutant BoΔ*wadA* had levels of reactivity similar to those of the parental strain (Figure 3A). In addition, the binding of anti-Omp MAbs to both BoΔ*wadB* and BoΔ*wadC* mutants revealed significant differences compared to the parental strain: while BoΔ*wadB* reacted more strongly with MAbs against the major Omps Omp25, Omp31 and also the lipoproteins Omp16 and Omp19, BoΔ*wadC* had decreased reactivity with all the anti-Omp MAbs (Figure 3B). Similarly, the anti-Omp2b MAbs A68/25G05/A05 and A68/15B06/C08 bound more strongly to BoΔ*wadB* than parental *B. ovis* PA, and very low levels of reactivity with Bo∆*wadC*; however, the anti-Omp2b MAb A63/04D11/G01 displayed increased binding to Bo∆*wadC* and a low binding to BoΔ*wadB*. These results suggest that the defects in the LPS oligosaccharide of both mutants are different and affect the exposure and/or conformation of the major Omps also in a different way. The MAb reactivity of the major Omps in the BoΔ*wadA* mutant was similar to that of the parental strain in all the MAbs tested except the antibody directed against Omp31 A59/10F09/G10 which showed an increased reactivity (Figure 3B).

**Figure 4:**
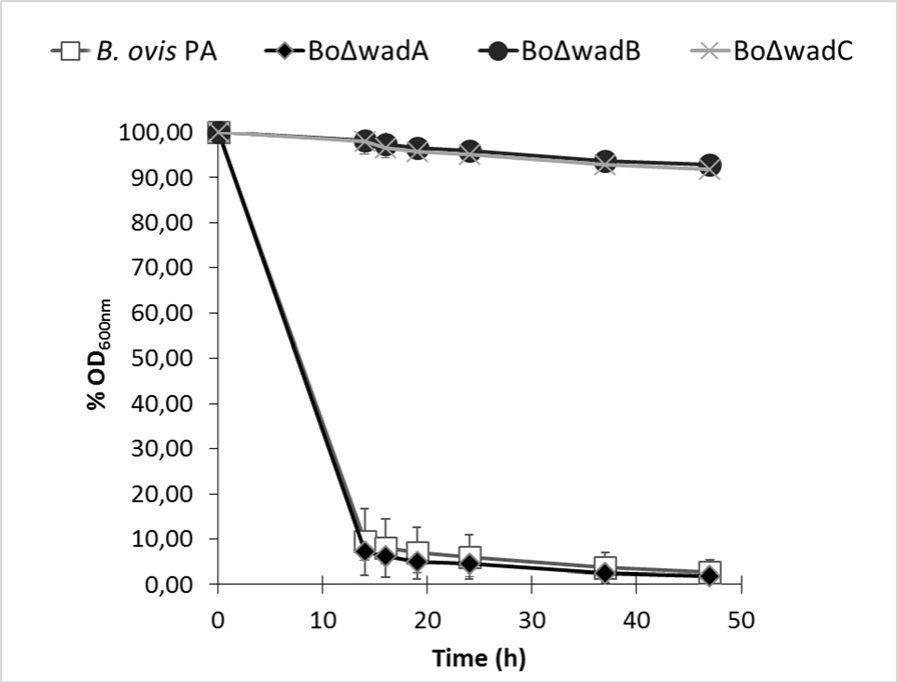
**Autoagglutination properties of** ***B. ovis*** **PA and the LPS mutants.** Results are the mean ± SD of three independent experiments. Sibling revertant strains behaved as the parental strain (data not shown).

**Figure 5:**
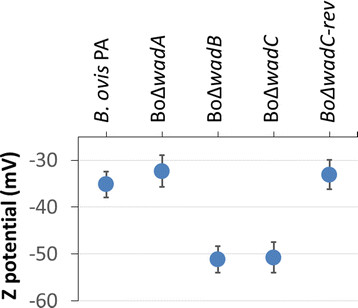
**Surface charge (Z potential) of** ***B. ovis*** **and the LPS mutants.** Results are the mean ± SD of ten independent experiments. BoΔ*wadC-rev* is the sibling revertant strain of the BoΔ*wadC* mutant, and the same results were obtained with BoΔ*wadB-rev* (not shown).

Autoagglutination is a property characteristic of some *Brucella* R mutants. Parental *B. ovis* PA, BoΔ*wadA* and all the sibling revertant strains showed autoagglutination, with a 90% reduction of the initial OD_600_ value in a few hours. On the contrary, mutants BoΔ*wadB* and BoΔ*wadC* remained in suspension for at least 48 h (Figure 4). This effect correlated with an increase in negative surface charge (Zeta potential, Figure 5). We also studied whether the resistance to complement-mediated killing in normal serum typical of brucellae was affected by the mutations [[36]]. We found that, whereas the parental strain *B. ovis* PA, BoΔ*wadA* and the sibling revertant strain were resistant to non-immune serum (survival percentages of 93.0 ± 5.5, 92.0 ± 4.8, 101 ± 6.9, respectively), BoΔ*wadB* and BoΔ*wadC* mutants were significantly (*p* < 0.05) affected (survival percentages of 74.0 ± 3.9 and 68.0 ± 3.2, respectively). On the contrary, polymyxin B resistance, another important envelope property of brucellae, was not significantly affected (not shown).

### Both BoΔ*wadB* and BoΔ*wadC* mutants are attenuated and protect against *B. ovis* PA infection in mice

The colonization and persistence of *B. ovis* LPS gene deletion mutants in the spleens of mice was evaluated at 3 and 10 weeks after intraperitoneal infection with 5–7 × 10^5^ CFU/mouse. While the infection produced by BoΔ*wadA* reached the levels of infection of *B. ovis* PA, mutants BoΔ*wadB* and BoΔ*wadC* showed significant (*p* < 0.005) attenuation (Table 3). In fact, the splenic concentrations reached by the latter mutants at 3 weeks post-infection (i.e. the time-point at which peak splenic concentrations are reached by virulent strains) were significantly (*p* < 0.005) lower than that reached by the parental strain. Thereafter, at 10 weeks post-infection, BoΔ*wadA* persisted at the levels of the parental strain while BoΔ*wadB* and BoΔ*wadC* mutants showed significantly (*p* < 0.005) reduced persistence, being practically cleared from the spleens in most of the inoculated mice (Table 3). Such significant attenuation was not attributable to the mutagenesis procedure, since both sibling revertant strains reached and maintained levels of splenic infections similar to those reached by the parental strain (Table 3).

**Table 3:** **Colonization of** ***B. ovis*** **PA and the LPS mutant strains in mouse spleens**

***B. ovis*** **strain**	**log**_**10**_**CFU in spleen**		
	**3 weeks post infection**	**10 weeks post infection**	
	**mean ± SD**	**mean ± SD**	**Cleared/Totals**
*B. ovis* PA	6.56 ± 0.52	4.67 ± 0.86	0/5
BoΔ*wadA*	6.78 ± 0.40	4.41 ± 2.01	0/5
BoΔ*wadB*	2.31 ± 1.91^a^	0.80 ± 0.42^a^	5/5^a^
BoΔ*wadC*	3.82 ± 0.72^a^	1.11 ± 0.77^a^	4/5^a^
BoΔ*wadB*-rev^b^	5.89 ± 0.77	4.90 ± 0.45	0/5
BoΔ*wadC*-rev^b^	6.49 ± 0.23	4.71 ± 1.68	0/5

Statistical comparison (*n* = 5) of mean log CFU/spleen or percentage of mice found free from infection in spleens by totals, respectively: ^a^*P* < 0.005 vs. *B. ovis* PA; ^b^Sibling revertant mutants.

The efficacy of BoΔ*wadB* and BoΔ*wadC* as vaccines was studied in mice challenged with *B. ovis* PA. As shown in Table 4, these two mutants conferred significant protection with respect to the unvaccinated controls. However, the protection conferred by BoΔ*wadB* was the most effective one, improving significantly (*p* < 0.05) that conferred by others at standard conditions, and even preventing the virulent infection in 7 out 10 mice (Table 4).

**Table 4:** **Efficacy of** ***B. ovis*** **LPS mutants in BALB/c mice against a virulent** ***B. ovis*** **infection**

**Vaccine (dose/route)**	**Log**_**10**_**CFU in spleen (mean ± SD)**	**No. of non-infected/ total mice studied**
BoΔ*wadB* (10^8^/IP)	1.08 ± 1.22^a,c^	7/10^a,c^
BoΔ*wadC* (10^8^/IP)	2.18 ± 1.59^a^	5/10^b^
*B. melitensis* Rev 1 (10^5^/SC)	2.75 ± 1.26^b^	2/10
PBS (unvaccinated control)	6.24 ± 0.48^c^	0/10

^a^*p* < 0.005 and ^, b^*p* < 0.05 vs. unvaccinated control; ^c^*p* < 0.05 vs. Rev 1 control.

## Discussion

Several differences have been shown in the structure of the LPS among the different species of the genus *Brucella* [[37]–[40]]. Wild-type *B. melitensis*, *B. abortus*, *B. suis*, *B. neotomae*, *B. ceti*, *B. pinnipedialis* and *B. microti* produce a S-LPS where the O-polysaccharide is linked to the core oligosaccharide. However, *B. ovis* and *B. canis* lack this O-polysaccharide thus producing an R-LPS. The genetic bases of this R phenotype have been partially described [[41]]. In *B. ovis*, the genome shows a 15 Kb deletion which includes genes *wboA* and *wboB* [[41],[42]], both involved in the synthesis of the O-polysaccharide, and several point mutations generating nonfunctional proteins, *wbkF* and *wzt* among others [[43]], involved in bactoprenol priming (see also below) and export of the O-polysaccharide to the periplasm, respectively.

The structures of the core and repeated O-polysaccharide fragments of *B. abortus* biovar 4, *B. melitensis* biovar 3 and *B. suis* biovar 4 obtained after acid hydrolysis of S-LPS have been recently published [[8]]. The structure available for the core oligosaccharide (Figure 1) shows the existence of a side oligosaccharide chain composed of four residues of glucosamine, plus glucose and mannose, the latter linked to Kdo I, with Kdo II linked to another glucose from which the O-polysaccharide stems. Although the precise structure of LPS of *B. ovis* remains to be determined, a previous immunochemical assay carried out with R-LPS extracted from *B. ovis* REO198 found fragments that were estimated to contain 2, 4, 6 and 7 sugars, including glucose, mannose, glucosamine, Kdo and an unidentified sugar [[44]]. Up to now only three glycosyltransferases have been shown to be involved in the synthesis of the LPS core in *B. abortus* and *B. melitensis* (*wadA*, *wadB* and *wadC*), and *B. ovis* carries the corresponding orthologous ORF. Although the homology of these glycosyltransferases suggests that the composition of *B. ovis* core LPS is similar to other *Brucella* species, antigenic and immunochemical analyses of LPS extracts have showed differences [[3],[45]].

In *B. abortus* and *B. melitensis*, the deletion of *wadA* results in an R phenotype and an altered LPS core [[17],[18]]. In *B. melitensis*, the *wadA* mutant carries a defective LPS core but also synthesizes a free cytoplasmic O-polysaccharide, which strongly suggests that *wadA* codes for a glycosyltransferase involved in the synthesis of the core section linked to the O-polysaccharide [[18]]. Taking into account previous electrophoretic analysis of the R-LPS, *wadA* is likely to encode the enzyme involved in transferring the glucose linked to quinovosamine [[18]] (Figure 1). In the case of *B. ovis*, the reactivity with MAbs, the cell surface properties, the electrophoretic analyses of the LPS and the virulence assays all showed a similar behavior for mutant BoΔ*wadA* and the parental strain. Quinovosamine, absent in *B. ovis* REO 198 LPS [[45]], is in all likelihood the sugar priming bactoprenol for O-polysaccharide synthesis [[18]], and *B. ovis* carries mutations in *wbkF*, involved in bactoprenol priming, so LPS oligosaccharide lacks a terminal glucose in the *wadA* mutants. Such a small defect would be consistent with the fact that the *B. ovis* Δ*wadA* mutant was practically indistinguishable from the parental strain.

Although the LPS core of *B. abortus* and *B. melitensis* mutants is altered, the O-polysaccharide remains in place [5–7 and Conde R., Arce-Gorvel V., Iriarte M., Gorvel J.P. and Moriyón I., unpublished results]. This evidence shows that glycosyltransferases WadB and WadC are responsible for the synthesis of the glucosamine rich oligosaccharide stemming from Kdo II (Figure 1). Specifically, it has been proposed that WadC could be implicated in the transfer of the first mannose to the lateral chain, while WadB would transfer other sugar [[6],[7]]. In *B. abortus*, both mutants induce a pro-inflammatory response higher than that of wild-type bacteria and are thus attenuated in dendritic cells and mice [[5]–[7]]. Moreover, they are more sensitive to the bactericidal action of the nonimmune serum and cationic peptides [[5],[7]]. It has also been proved that the core defect enhances recognition by the TLR4-MD2 receptor/co-receptor system and, accordingly, a glucosamine-rich branch of the LPS core of the *B. abortus* and *B. melitensis* acts as a shield against recognition by several innate immune systems, thus representing a virulence mechanism of these *Brucella* spp. [[5]]. In *B. ovis*, the LPS of the mutants Δ*wadB* and Δ*wadC* lacks a part of the core oligosaccharide (component I in Figure 2) and the defects generate a marked increase in the negative value of zeta potential that can be explained by the loss of all or part of the positively charged glucosamine residues (Figure 1). The attenuation observed for mutants BoΔ*wadB* and BoΔ*wadC* show for the first time that the LPS of a naturally R *Brucella* pathogenic species (i.e. *B. ovis*) is essential for survival in the mouse model. Even though the exact mechanism remains to be elucidated, the proposed core defect is likely to facilitate the recognition of *B. ovis* LPS by innate immunity. Indeed, this hypothesis is supported by the above-summarized results obtained with the homologous *B. abortus* and *B. melitensis* mutants and with the fact that, rather than the O-polysaccharide, it is the lipid A-core overall structure that is recognized by multiple innate immunity systems [[5]–[7],[17],[18]]. Nevertheless, other effects cannot be disregarded because the mutants BoΔ*wadB* and BoΔ*wadC* were affected not only in the LPS but also in the topology of the outer membrane as detected with anti-Omp MAbs. Little is known about how these changes may affect the virulence of brucellae but it has been shown that not all the *B. melitensis* R mutants are equivalent in their surface properties and outer membrane topology, and that they have different levels of interaction with host cells, virulence and vaccination power [[12],[18]].

Recently, Conde-Álvarez et al. [[6]] proposed a new strategy to develop vaccines against brucellosis based on the concept that the surface molecules of *Brucella* effectively evade a prompt detection by innate immunity [[46]], and that surface changes such as those introduced by mutations affecting the glucosamine branch of the core would overcome hampered recognition thus producing a protective Th1 response. This concept was based on a comparison of a *B. abortus wadC* mutant and the current vaccine *B. abortus* S19 in the mouse model [[6]]. Although we have not evaluated the immune response in this work, our results add further weight to the value of this strategy and show the possibility of obtaining vaccines against brucellosis caused by *B. ovis* that could be used in areas free of *B. melitensis*. However, a potential defect of these vaccines would be their interference in the diagnosis of *B. ovis*, which is carried out using tests that detect antibodies to both Omp and R-LPS [[47]]. These problems, however, could be circumvented by the use of antigenically-tagged brucellosis vaccines, a strategy recently shown to be effective for the vaccine *B. abortus* S19 in the mouse model [[48]]. Research is in progress to evaluate all these possibilities in sheep.

## Competing interests

MI and IM are co-owners of the patent “Modified Gram-negative bacteria for use as vaccines” (N°PCT/EP2010/063921 [WO2011/033129]) that covers the use of *Brucella* LPS core genes.

## Authors’ contributions

PS-L carried out the experiments and participated in the design of the study and draft of the manuscript. YG-R, AZ, MI, RC-A and NV collaborated with the design and construction of the mutants. MZ and AC helped with the immunochemical studies and the characterization of the LPS. M-JG, AZ-B and BSR participated in mice in vivo studies. IL-G and IM conceived the study, participated in its design, coordinated the work and helped to draft the manuscript. All authors read and approved the final manuscript.

## Additional file

## Supplementary Material

Additional file 1: **Confirmation of the construction of the mutants.** A: schematic representation of the ORF indicating in grey colour the deleted region and the primers used to check the strains. B: For each gene, two confirmation PCR assays were carried out with specific primers. Primers F1-R4 flank the ORF and show different sizes between the mutant and the wild type gene; and primers F1-R5 cover from upstream of the ORF to the deleted region, so that only strains carrying the complete ORF will be amplified and the specific amplicon will be shown. (1) 1 kb plus ladder marker; (2) *B. ovis* PA; (3) *B. ovis* deleted mutant; (4) *B. ovis* sibling revertant strain. Click here for file
