# Supplementary figures and images for: Mutants in the lipopolysaccharide of Brucella ovis are attenuated and protect against B. ovis infection in mice
Source: Vet Res. 2014 Jul 17;45(1):72. doi: 10.1186/s13567-014-0072-0 (PMC4107470; doi:10.1186/s13567-014-0072-0)

A

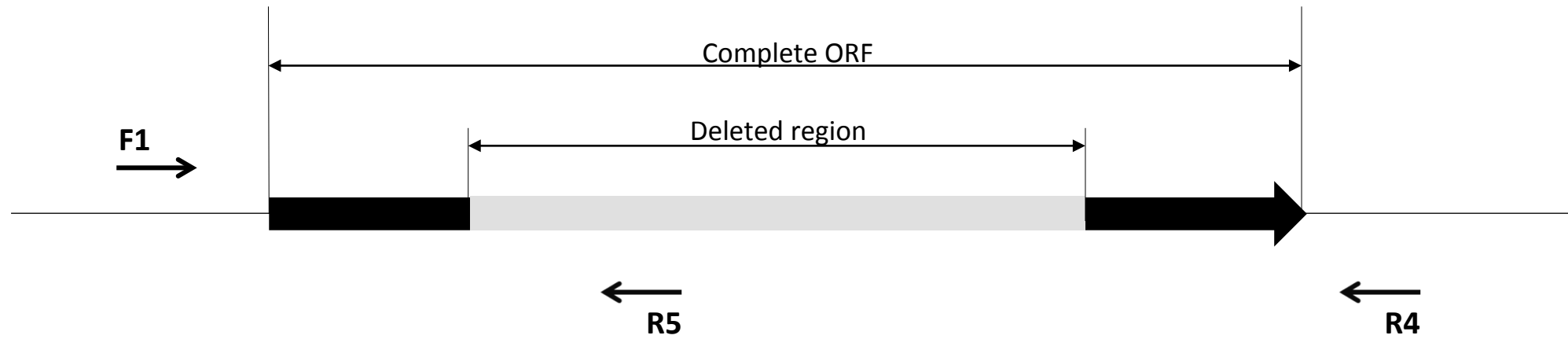

B

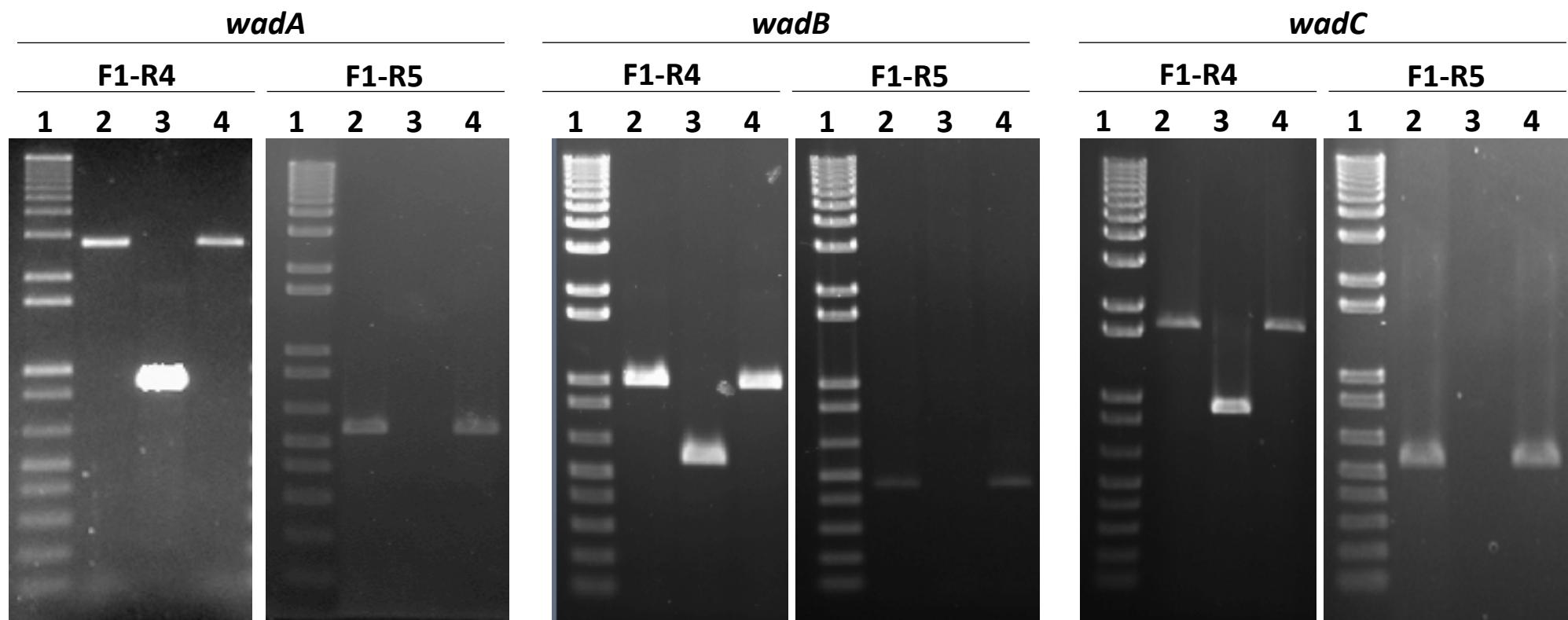

Supplement: Additional file 1: — Confirmation of the construction of the mutants. A: schematic representation of the ORF indicating in grey colour the deleted region and the primers used to check the strains. B: For each gene, two confirmation PCR assays were carried out with specific primers. Primers F1-R4 flank the ORF and show different sizes between the mutant and the wild type gene; and primers F1-R5 cover from upstream of the ORF to the deleted region, so that only strains carrying the complete ORF will be amplified and the specific amplicon will be shown. (1) 1 kb plus ladder marker; (2) B. ovis PA; (3) B. ovis deleted mutant; (4) B. ovis sibling revertant strain. [file s13567-014-0072-0-S1.pdf]
